# Supplementary material for: Modeling glioblastoma heterogeneity as a dynamic network of cell states
Source: Mol Syst Biol. 2021 Sep 16;17(9):e10105. doi: 10.15252/msb.202010105 (PMC8444284; doi:10.15252/msb.202010105)
Supplement: Supplementary file 6 — Source Data for Figure 5 [file MSB-17-e10105-s004.zip › Figure5A_sourcedata/GSEA_3071/hallmarks_stateV.GseaPreranked.1623742546329/HALLMARK_E2F_TARGETS.html]

Details for gene set HALLMARK\_E2F\_TARGETS[GSEA]

|  || Dataset | state43071 |
| Phenotype | NoPhenotypeAvailable |
| Upregulated in class | na\_pos |
| GeneSet | HALLMARK\_E2F\_TARGETS |
| Enrichment Score (ES) | 0.6596706 |
| Normalized Enrichment Score (NES) | 3.9468064 |
| Nominal p-value | 0.0 |
| FDR q-value | 0.0 |
| FWER p-Value | 0.0 |
Table: GSEA Results Summary

  

Fig 1: Enrichment plot: HALLMARK\_E2F\_TARGETS      
 Profile of the Running ES Score & Positions of GeneSet Members on the Rank Ordered List

  

| PROBE | GENE SYMBOL | GENE\_TITLE | RANK IN GENE LIST | RANK METRIC SCORE | RUNNING ES | CORE ENRICHMENT || 1 | ATAD2 |  |  | 2 | 2.214 | 0.0149 | Yes |
| 2 | HMGB2 |  |  | 5 | 2.011 | 0.0284 | Yes |
| 3 | CCP110 |  |  | 9 | 1.920 | 0.0410 | Yes |
| 4 | PCNA |  |  | 10 | 1.908 | 0.0542 | Yes |
| 5 | TOP2A |  |  | 14 | 1.842 | 0.0663 | Yes |
| 6 | MCM4 |  |  | 17 | 1.815 | 0.0784 | Yes |
| 7 | RAD51AP1 |  |  | 22 | 1.757 | 0.0898 | Yes |
| 8 | RRM2 |  |  | 34 | 1.660 | 0.0991 | Yes |
| 9 | MYBL2 |  |  | 35 | 1.651 | 0.1105 | Yes |
| 10 | MKI67 |  |  | 45 | 1.598 | 0.1197 | Yes |
| 11 | BRCA1 |  |  | 46 | 1.592 | 0.1307 | Yes |
| 12 | HELLS |  |  | 48 | 1.588 | 0.1415 | Yes |
| 13 | MCM3 |  |  | 49 | 1.586 | 0.1524 | Yes |
| 14 | EZH2 |  |  | 55 | 1.534 | 0.1620 | Yes |
| 15 | MXD3 |  |  | 62 | 1.500 | 0.1712 | Yes |
| 16 | E2F8 |  |  | 67 | 1.488 | 0.1807 | Yes |
| 17 | CDK1 |  |  | 69 | 1.488 | 0.1907 | Yes |
| 18 | SPC25 |  |  | 76 | 1.442 | 0.1995 | Yes |
| 19 | UBE2T |  |  | 79 | 1.432 | 0.2090 | Yes |
| 20 | MCM7 |  |  | 88 | 1.400 | 0.2171 | Yes |
| 21 | DEK |  |  | 94 | 1.384 | 0.2256 | Yes |
| 22 | POLD3 |  |  | 111 | 1.340 | 0.2318 | Yes |
| 23 | CENPM |  |  | 114 | 1.337 | 0.2406 | Yes |
| 24 | TACC3 |  |  | 116 | 1.334 | 0.2496 | Yes |
| 25 | SMC4 |  |  | 127 | 1.320 | 0.2568 | Yes |
| 26 | BARD1 |  |  | 132 | 1.310 | 0.2650 | Yes |
| 27 | MMS22L |  |  | 134 | 1.306 | 0.2738 | Yes |
| 28 | LIG1 |  |  | 145 | 1.284 | 0.2807 | Yes |
| 29 | NCAPD2 |  |  | 157 | 1.252 | 0.2872 | Yes |
| 30 | TMPO |  |  | 161 | 1.243 | 0.2952 | Yes |
| 31 | CDKN3 |  |  | 163 | 1.239 | 0.3036 | Yes |
| 32 | LMNB1 |  |  | 165 | 1.238 | 0.3119 | Yes |
| 33 | DNMT1 |  |  | 166 | 1.237 | 0.3204 | Yes |
| 34 | CDCA3 |  |  | 169 | 1.230 | 0.3285 | Yes |
| 35 | NASP |  |  | 180 | 1.212 | 0.3349 | Yes |
| 36 | KIF4A |  |  | 191 | 1.193 | 0.3412 | Yes |
| 37 | USP1 |  |  | 202 | 1.168 | 0.3473 | Yes |
| 38 | KIF22 |  |  | 213 | 1.157 | 0.3533 | Yes |
| 39 | CDKN2C |  |  | 223 | 1.143 | 0.3595 | Yes |
| 40 | ASF1B |  |  | 237 | 1.130 | 0.3647 | Yes |
| 41 | MCM2 |  |  | 238 | 1.130 | 0.3725 | Yes |
| 42 | RFC3 |  |  | 239 | 1.130 | 0.3803 | Yes |
| 43 | MCM5 |  |  | 243 | 1.127 | 0.3875 | Yes |
| 44 | SMC1A |  |  | 251 | 1.120 | 0.3939 | Yes |
| 45 | RAD21 |  |  | 256 | 1.109 | 0.4007 | Yes |
| 46 | TIMELESS |  |  | 259 | 1.105 | 0.4080 | Yes |
| 47 | BRCA2 |  |  | 274 | 1.089 | 0.4127 | Yes |
| 48 | MCM6 |  |  | 282 | 1.080 | 0.4188 | Yes |
| 49 | AURKB |  |  | 297 | 1.068 | 0.4235 | Yes |
| 50 | STMN1 |  |  | 307 | 1.062 | 0.4290 | Yes |
| 51 | PSIP1 |  |  | 313 | 1.053 | 0.4353 | Yes |
| 52 | DUT |  |  | 314 | 1.050 | 0.4426 | Yes |
| 53 | WEE1 |  |  | 322 | 1.044 | 0.4484 | Yes |
| 54 | RFC2 |  |  | 337 | 1.025 | 0.4527 | Yes |
| 55 | POLE |  |  | 344 | 1.023 | 0.4586 | Yes |
| 56 | GINS4 |  |  | 359 | 0.997 | 0.4628 | Yes |
| 57 | CDKN1B |  |  | 360 | 0.997 | 0.4697 | Yes |
| 58 | CDKN1A |  |  | 363 | 0.993 | 0.4761 | Yes |
| 59 | PSMC3IP |  |  | 382 | 0.968 | 0.4793 | Yes |
| 60 | GINS1 |  |  | 385 | 0.965 | 0.4855 | Yes |
| 61 | CIT |  |  | 391 | 0.958 | 0.4912 | Yes |
| 62 | RPA3 |  |  | 395 | 0.953 | 0.4972 | Yes |
| 63 | SLBP |  |  | 403 | 0.949 | 0.5023 | Yes |
| 64 | SPC24 |  |  | 414 | 0.938 | 0.5069 | Yes |
| 65 | DLGAP5 |  |  | 418 | 0.936 | 0.5127 | Yes |
| 66 | MAD2L1 |  |  | 421 | 0.932 | 0.5188 | Yes |
| 67 | UBR7 |  |  | 423 | 0.932 | 0.5250 | Yes |
| 68 | DSCC1 |  |  | 427 | 0.926 | 0.5308 | Yes |
| 69 | KPNA2 |  |  | 435 | 0.919 | 0.5358 | Yes |
| 70 | CENPE |  |  | 437 | 0.918 | 0.5419 | Yes |
| 71 | LBR |  |  | 438 | 0.916 | 0.5482 | Yes |
| 72 | RACGAP1 |  |  | 443 | 0.912 | 0.5537 | Yes |
| 73 | ORC6 |  |  | 449 | 0.908 | 0.5590 | Yes |
| 74 | CCNE1 |  |  | 461 | 0.901 | 0.5631 | Yes |
| 75 | SPAG5 |  |  | 481 | 0.884 | 0.5655 | Yes |
| 76 | UBE2S |  |  | 514 | 0.854 | 0.5652 | Yes |
| 77 | DCK |  |  | 516 | 0.854 | 0.5708 | Yes |
| 78 | PLK1 |  |  | 526 | 0.850 | 0.5750 | Yes |
| 79 | BUB1B |  |  | 535 | 0.846 | 0.5792 | Yes |
| 80 | BIRC5 |  |  | 541 | 0.842 | 0.5841 | Yes |
| 81 | TCF19 |  |  | 542 | 0.842 | 0.5899 | Yes |
| 82 | PRKDC |  |  | 554 | 0.828 | 0.5934 | Yes |
| 83 | CDC25B |  |  | 566 | 0.820 | 0.5969 | Yes |
| 84 | DIAPH3 |  |  | 585 | 0.808 | 0.5990 | Yes |
| 85 | TK1 |  |  | 591 | 0.806 | 0.6036 | Yes |
| 86 | TRA2B |  |  | 596 | 0.802 | 0.6083 | Yes |
| 87 | STAG1 |  |  | 602 | 0.800 | 0.6129 | Yes |
| 88 | HMMR |  |  | 622 | 0.788 | 0.6146 | Yes |
| 89 | CDC25A |  |  | 631 | 0.782 | 0.6185 | Yes |
| 90 | HNRNPD |  |  | 659 | 0.769 | 0.6185 | Yes |
| 91 | KIF18B |  |  | 698 | 0.747 | 0.6162 | Yes |
| 92 | UNG |  |  | 703 | 0.745 | 0.6206 | Yes |
| 93 | SMC3 |  |  | 708 | 0.743 | 0.6249 | Yes |
| 94 | PLK4 |  |  | 720 | 0.739 | 0.6279 | Yes |
| 95 | ANP32E |  |  | 722 | 0.739 | 0.6328 | Yes |
| 96 | CTCF |  |  | 729 | 0.731 | 0.6367 | Yes |
| 97 | AURKA |  |  | 761 | 0.718 | 0.6356 | Yes |
| 98 | TUBG1 |  |  | 776 | 0.712 | 0.6378 | Yes |
| 99 | CHEK1 |  |  | 779 | 0.709 | 0.6423 | Yes |
| 100 | CBX5 |  |  | 783 | 0.708 | 0.6466 | Yes |
| 101 | TRIP13 |  |  | 804 | 0.698 | 0.6475 | Yes |
| 102 | RBBP7 |  |  | 829 | 0.687 | 0.6476 | Yes |
| 103 | TUBB |  |  | 836 | 0.685 | 0.6511 | Yes |
| 104 | RNASEH2A |  |  | 859 | 0.674 | 0.6515 | Yes |
| 105 | CHEK2 |  |  | 882 | 0.667 | 0.6518 | Yes |
| 106 | MELK |  |  | 915 | 0.653 | 0.6501 | Yes |
| 107 | PTTG1 |  |  | 936 | 0.647 | 0.6506 | Yes |
| 108 | CKS2 |  |  | 966 | 0.634 | 0.6493 | Yes |
| 109 | PRIM2 |  |  | 968 | 0.633 | 0.6535 | Yes |
| 110 | POLD1 |  |  | 998 | 0.620 | 0.6521 | Yes |
| 111 | RANBP1 |  |  | 999 | 0.620 | 0.6564 | Yes |
| 112 | CDCA8 |  |  | 1043 | 0.610 | 0.6522 | Yes |
| 113 | MLH1 |  |  | 1045 | 0.610 | 0.6562 | Yes |
| 114 | DONSON |  |  | 1082 | 0.597 | 0.6533 | Yes |
| 115 | SRSF2 |  |  | 1084 | 0.597 | 0.6573 | Yes |
| 116 | MSH2 |  |  | 1107 | 0.590 | 0.6571 | Yes |
| 117 | PDS5B |  |  | 1140 | 0.580 | 0.6548 | Yes |
| 118 | HMGB3 |  |  | 1154 | 0.577 | 0.6563 | Yes |
| 119 | POLA2 |  |  | 1161 | 0.574 | 0.6591 | Yes |
| 120 | DEPDC1 |  |  | 1179 | 0.569 | 0.6597 | Yes |
| 121 | EXOSC8 |  |  | 1230 | 0.553 | 0.6537 | No |
| 122 | RAD1 |  |  | 1234 | 0.552 | 0.6570 | No |
| 123 | CSE1L |  |  | 1259 | 0.543 | 0.6560 | No |
| 124 | RPA1 |  |  | 1307 | 0.530 | 0.6505 | No |
| 125 | KIF2C |  |  | 1309 | 0.530 | 0.6540 | No |
| 126 | MRE11 |  |  | 1335 | 0.523 | 0.6527 | No |
| 127 | CCNB2 |  |  | 1373 | 0.513 | 0.6491 | No |
| 128 | WDR90 |  |  | 1381 | 0.511 | 0.6512 | No |
| 129 | NUP107 |  |  | 1388 | 0.509 | 0.6536 | No |
| 130 | SHMT1 |  |  | 1417 | 0.499 | 0.6516 | No |
| 131 | PPM1D |  |  | 1419 | 0.498 | 0.6548 | No |
| 132 | RFC1 |  |  | 1499 | 0.480 | 0.6427 | No |
| 133 | CKS1B |  |  | 1502 | 0.479 | 0.6456 | No |
| 134 | POP7 |  |  | 1591 | 0.461 | 0.6317 | No |
| 135 | SSRP1 |  |  | 1634 | 0.451 | 0.6266 | No |
| 136 | ILF3 |  |  | 1698 | 0.436 | 0.6173 | No |
| 137 | ESPL1 |  |  | 1733 | 0.430 | 0.6137 | No |
| 138 | DCTPP1 |  |  | 1796 | 0.418 | 0.6045 | No |
| 139 | ING3 |  |  | 1844 | 0.411 | 0.5982 | No |
| 140 | SMC6 |  |  | 1867 | 0.407 | 0.5967 | No |
| 141 | EED |  |  | 1934 | 0.399 | 0.5866 | No |
| 142 | PNN |  |  | 2076 | 0.376 | 0.5617 | No |
| 143 | XPO1 |  |  | 2091 | 0.374 | 0.5616 | No |
| 144 | PA2G4 |  |  | 2112 | 0.372 | 0.5602 | No |
| 145 | LUC7L3 |  |  | 2142 | 0.369 | 0.5571 | No |
| 146 | NUP205 |  |  | 2169 | 0.366 | 0.5546 | No |
| 147 | PAICS |  |  | 2403 | 0.334 | 0.5115 | No |
| 148 | SRSF1 |  |  | 2505 | 0.322 | 0.4940 | No |
| 149 | RPA2 |  |  | 2570 | 0.314 | 0.4837 | No |
| 150 | JPT1 |  |  | 2712 | 0.298 | 0.4583 | No |
| 151 | GINS3 |  |  | 2847 | 0.284 | 0.4342 | No |
| 152 | CNOT9 |  |  | 2951 | 0.274 | 0.4160 | No |
| 153 | RAD50 |  |  | 3331 | -0.272 | 0.3440 | No |
| 154 | NBN |  |  | 3377 | -0.278 | 0.3372 | No |
| 155 | TBRG4 |  |  | 3442 | -0.288 | 0.3267 | No |
| 156 | NME1 |  |  | 3615 | -0.317 | 0.2954 | No |
| 157 | EIF2S1 |  |  | 3809 | -0.351 | 0.2602 | No |
| 158 | MTHFD2 |  |  | 4212 | -0.444 | 0.1850 | No |
| 159 | CDKN2A |  |  | 4301 | -0.472 | 0.1711 | No |
| 160 | LYAR |  |  | 4584 | -0.578 | 0.1201 | No |
| 161 | PRDX4 |  |  | 4821 | -0.732 | 0.0792 | No |
| 162 | HMGA1 |  |  | 4962 | -0.878 | 0.0580 | No |
| 163 | MYC |  |  | 5020 | -0.972 | 0.0536 | No |
Table: GSEA details [plain text format]

  

Fig 2: HALLMARK\_E2F\_TARGETS: Random ES distribution      
 Gene set null distribution of ES for **HALLMARK\_E2F\_TARGETS**
